# Computational approach to discriminate human and mouse sequences in patient-derived tumour xenografts

**DOI:** 10.1186/s12864-017-4414-y

**Published:** 2018-01-05

**Authors:** Maurizio Callari, Ankita Sati Batra, Rajbir Nath Batra, Stephen-John Sammut, Wendy Greenwood, Harry Clifford, Colin Hercus, Suet-Feung Chin, Alejandra Bruna, Oscar M. Rueda, Carlos Caldas

**Affiliations:** 1 CRUK Cambridge Institute and Department of Oncology, University of Cambridge, Li Ka Shing Centre, Robinson Way, Cambridge, CB2 0RE UK; 2 Novocraft Technologies Sdn Bhd, C-23A-05, 3 Two Square, Jalan 19/1, Section 19, 46300 Petaling Jaya, Selangor Darul Ehsan Malaysia

**Keywords:** In silico combined human-mouse reference genome, High throughput sequencing, Short-reads, Alignment, ICRG, Mouse stroma, Patient-derived tumour xenografts

## Abstract

**Background:**

Patient-Derived Tumour Xenografts (PDTXs) have emerged as the pre-clinical models that best represent clinical tumour diversity and intra-tumour heterogeneity. The molecular characterization of PDTXs using High-Throughput Sequencing (HTS) is essential; however, the presence of mouse stroma is challenging for HTS data analysis. Indeed, the high homology between the two genomes results in a proportion of mouse reads being mapped as human.

**Results:**

In this study we generated Whole Exome Sequencing (WES), Reduced Representation Bisulfite Sequencing (RRBS) and RNA sequencing (RNA-seq) data from samples with known mixtures of mouse and human DNA or RNA and from a cohort of human breast cancers and their derived PDTXs. We show that using an In silico Combined human-mouse Reference Genome (ICRG) for alignment discriminates between human and mouse reads with up to 99.9% accuracy and decreases the number of false positive somatic mutations caused by misalignment by >99.9%. We also derived a model to estimate the human DNA content in independent PDTX samples. For RNA-seq and RRBS data analysis, the use of the ICRG allows dissecting computationally the transcriptome and methylome of human tumour cells and mouse stroma. In a direct comparison with previously reported approaches, our method showed similar or higher accuracy while requiring significantly less computing time.

**Conclusions:**

The computational pipeline we describe here is a valuable tool for the molecular analysis of PDTXs as well as any other mixture of DNA or RNA species.

**Electronic supplementary material:**

The online version of this article (10.1186/s12864-017-4414-y) contains supplementary material, which is available to authorized users.

## Background

Patient-Derived Tumour Xenografts (PDTXs) are emerging as the pre-clinical models that best represent the diversity of clinical tumours and intra-tumour heterogeneity [1–3]. PDTXs have been shown to be robust models to study tumour progression and evolution, test new cancer drugs and drug combinations, and unravel drug resistance mechanisms, contributing to the aim of reducing the high attrition rate in cancer drug development [4–8].

In the era of cancer genomics and precision medicine, the molecular analysis of PDTXs is a central component of their characterization. High-Throughput Sequencing (HTS) is used to profile these models at the genomic, epigenomic and transciptomic levels. We and others have observed that after implanting human cancer tissue fragments into immuno-compromised mice, the human stroma is rapidly lost and replaced by mouse stromal cells [2, 9, 10]. This results in an unknown proportion of mouse cells incorporated into the xenograft. As a consequence, a proportion of the sequencing reads obtained by HTS will be of mouse origin. Given the high homology of human and mouse genomes, mouse reads can be wrongly aligned to the human genome, hampering downstream analyses and data interpretation.

Previous studies have tried to address this issue. Conway et al. developed Xenome, a tool able to classify sequencing reads belonging to two different species; the output is a set of FastQ files that still need to be aligned to the appropriate genome [11]. More recently, Ahdesmäki and colleagues presented Disambiguate, that takes as input two bam files obtained by aligning the same FastQ file to the two relevant genomes and then classifies each read based on the alignment scores [12]. Currently, this approach can be used only in combination with a specific set of aligners.

Here we present a computational approach to distinguish human and mouse reads in HTS data based on the use of an In silico Combined human-mouse Reference Genome (ICRG) in the alignment step. We demonstrated the accuracy of the approach using control samples and a set of matched human breast cancers and derived PDTXs. In a direct comparison with Disambiguate and Xenome, our approach was quicker while showing similar or higher accuracy.

## Results

### Optimizing sequence alignment using the ICRG

In PDTX samples, a proportion of HTS reads originated from mouse DNA could have high enough homology to be aligned to the HRG. We reasoned that using the ICRG as the reference should allow the alignment software to find, for those mouse DNA reads, a better alignment score on the mouse genome, since both genomes are available simultaneously to the aligner software. This could represent the basis to accurately distinguish human and mouse reads in HTS data originated from PDTX models.

**Fig. 1:**
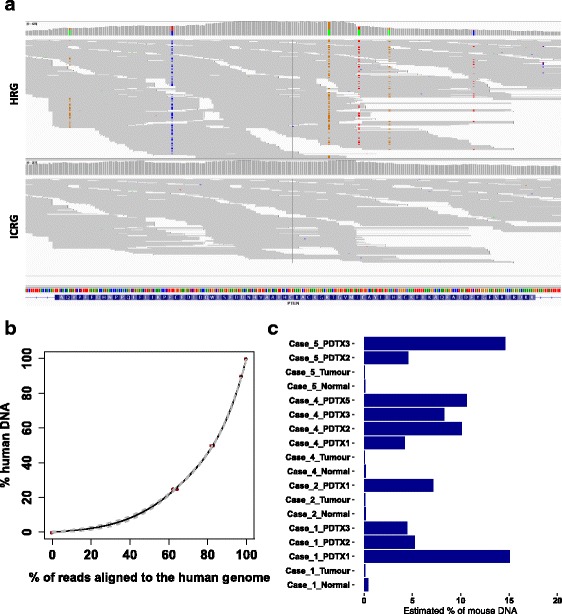
Use of the ICRG in WES data. **a** IGV plot of *PTEN* exon 5 (WES data from 25% human/75% mouse DNA sample). Top panel: bam files after alignment to the HRG. Bottom panel: bam files after alignment to the ICRG. Mismatching bases are highlighted using the corresponding colour code (A = green, C = blue, G = orange, T = red). **b** Correlation plot between the percentage of reads mapped to the human genome and percentage of human DNA content in the sample. The solid line shows the calibration curve fitted to the data using penalized regression splines and grey dashed lines show the standard error. **c** Prediction of mouse DNA content in primary human and PDTX samples using the calibration curve in (**b**)

To test this hypothesis, we performed WES in a dilution series containing known amounts of human and mouse DNA (Table 1). Sequencing data were aligned to both the HRG and the ICRG. In the 100% human DNA sample, a statistically significant decrease in alignment efficiency was observed when using the ICRG instead of the HRG (average efficiency = 90.48% for the HRG and 90.28% for the ICRG, paired t-test *P* = 0.009), however, the decrease in performance was negligible (0.2%). Aligning the WES data obtained by sequencing the 100% mouse DNA samples onto the HRG revealed that, on average, 6.9% of the reads were misaligned. As we show below, these misaligned reads are detrimental for downstream analyses. For example, a graphical representation of this misaligned mouse reads effect on a *PTEN* exon is shown in Fig. 1a. Mouse reads are wrongly aligned to the human genome if sequence similarity is high enough, but since identity is not 100%, all the mismatched bases could be called as false positive ‘somatic mutations’. In contrast, using the ICRG avoids this artefact altogether (Fig. 1a).

**Table 1:** Alignment of WES data from the human-mouse DNA dilution series

% of human DNA	%of mouse DNA	Replicate	Alignment efficiency human genome %	Alignment efficiency combined genome (%)	%of reads mapped on the human genome	%of reads mapped on the mouse genome	Estimated human DNA content
100	0	a	90.81	90.64	99.98	0.02	99.89
100	0	b	90.38	90.16	99.98	0.02	99.88
100	0	c	90.26	90.13	99.98	0.02	99.89
90	10	a	88.57	90.43	97.59	2.41	90.22
90	10	b	88.53	90.39	97.58	2.42	90.16
50	50	a	75.65	89.37	83.14	16.86	50.73
50	50	b	74.90	89.71	82.18	17.82	49.01
50	50	c	75.41	89.55	82.78	17.22	50.07
25	75	a	58.07	89.25	62.44	37.56	23.97
25	75	b	60.25	89.48	64.58	35.42	26.17
0	100	a	7.01	88.77	0.10	99.90	0.00
0	100	b	7.17	88.56	0.10	99.90	0.00
0	100	c	6.65	88.95	0.08	99.92	0.00

To study systematically the effectiveness of using the ICRG as a reference genome, we computed the percentage of reads mapping to the human and mouse genomes. As shown in Table 1, more than 99.9% of the reads from the pure human DNA sample and from the pure mouse DNA sample mapped to the correct genome. In diluted human-mouse DNA samples, the percentage of reads mapped to the correct genome did not match perfectly the percentage of input human and mouse DNA. Nevertheless, there was a non-linear relationship between the percentage of reads mapped to the human genome and the fraction of human DNA in the sample (Fig. 1b). We hypothesized this resulted from the enrichment step during WES library preparation, since the capture probes used have been designed for human exons. A careful look at the data revealed that using a generalised additive model was able to accurately estimate the human DNA content (Table 1). This model was used to estimate the mouse DNA content (as 100 - human DNA content) in a set of matched samples (normal/tumour/PDTX) for which WES data was available (Additional file 1). The model estimate of mouse DNA content for all primary human samples was negligible while the estimate ranged between 4.2 and 15.0% in PDTX samples (Fig. 1c).

### Improvement of mutation calling in WES PDTX data using the ICRG

The analysis of PDTX WES data aims at the identification of somatic mutations. However, the presence of misaligned mouse reads is likely to increase the false positive mutation rate. Therefore, we quantified the problem and verified whether the use of the ICRG for sequence alignment could effectively overcome it.

**Fig. 2:**
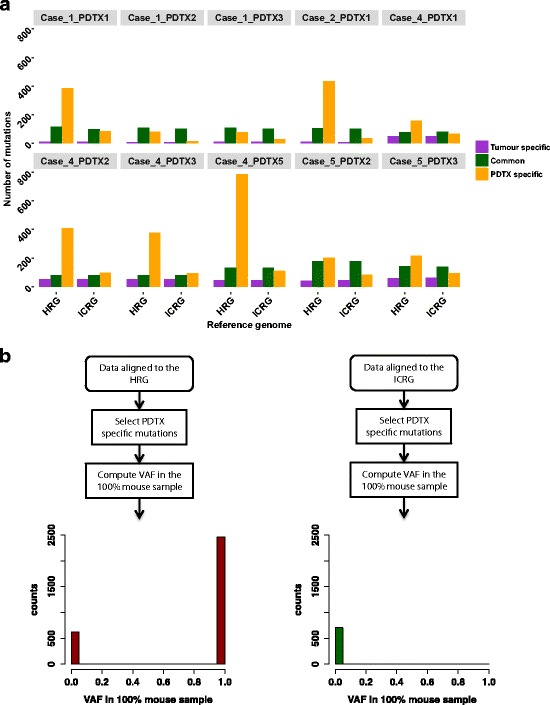
Impact of mouse reads on somatic mutation calling. **a** Bar plots showing numbers of somatic mutations identified in clinical tumours and matched PDTXs after alignment against either the HRG or the ICRG. Within each pair of clinical tumour and PDTX (*n* = 10), mutations were classified as ‘tumour specific’ (i.e. present in the tumour but not in the matched PDTX), ‘PDTX specific’ (i.e. present in the PDTX but not in the originating clinical tumour) and common (present in both tumour and PDTX). **b** Bar plots showing VAFs for all ‘PDTX specific’ mutations identified in the 10 pairs in (**a**) in the 100% mouse sample. Left panel- data aligned to the HRG; Right panel- data aligned to the ICRG

For each pair of PDTX and its originating clinical tumour, we identified the somatic mutations and quantified how many were present only in the tumour, only in the PDTX or in both. The analysis was performed on WES data aligned to either the HRG or the ICRG. Results, reported in Fig. 2a, show that the number of tumour specific mutations identified across 10 PDTX-clinical tumour pairs was not significantly affected by the reference genome used for alignment (average = 31.9 for the HRG and 32.5 for the ICRG, paired t-test *P* = 0.140). Similarly, no significant difference was observed for the common mutations (average = 113.1 for the HRG and 109.9 for the ICRG, paired t-test *P* = 0.086). In contrast, the number of PDTX specific mutations was high when the HRG was used and decreased dramatically by using the ICRG in the alignment step (average = 306.1 using HRG and 68.8 using ICRG, paired t-test *P* = 0.004).

To quantify the percentage of PDTX specific mutations caused by misalignment of mouse reads, we computed their VAF in one of the 100% mouse samples (replicate c in Table 1). As before, we repeated the analysis after alignment to the HRG or the ICRG (Fig. 2b). Of the 3123 PDTX specific mutations identified in the 10 PDTX-clinical tumour pairs aligned to the HRG, 2496 (79.9%) were present in the 100% mouse sample and are therefore false positives caused by the presence of mouse reads. Strikingly, only 712 PDTX specific mutations were identified in WES data aligned to the ICRG and only two of them were caused by mouse reads misalignment (Fig. 2b). In conclusion, the use of the ICRG was effective in removing >99.9% of false positive mutations caused by misaligned mouse reads.

The number of false positives caused by mouse reads is mostly a result of the mouse DNA content and sequencing depth. We noticed that even a small content of mouse DNA could generate a large number of false positive mutation calls. As an example, in Case_2_PDX1, sequenced with 69× coverage and with an estimated 7.2% mouse DNA content (Fig. 1c) a total of 405 false positive mutations specifically caused by misaligned mouse reads were identified when data were aligned to the HRG.

### Using the ICRG in the analysis of RRBS data

We next tested the usefulness of the ICRG in RRBS data analysis. To this aim, we profiled a set of normal, tumour and PDTX samples (Additional file 1). As shown in Table 2, in clinical samples the alignment efficiency was not affected by the reference genome used (average efficiency = 72.2 for the HRG and 72.4 for the ICRG, paired t-test *P* = 0.319). In contrast, the overall alignment efficiency increased in PDTX samples when the ICRG was used (average efficiency = 67.8 for the HRG and 72.7 for the ICRG, paired t-test *P* = 0.010). Such increase can be explained by the fact that the aligner could map most of the mouse reads present in the samples when the ICRG was used. In clinical samples, >98.8% of the reads were correctly mapped to the human genome (at least 99.9% in all but one). In contrast, in PDTXs, 2.6–23.1% of the reads mapped to the mouse genome (Table 2).

**Table 2:** Alignment of RRBS data and CpG quantification

Sampler	HRG		ICRG					Common human CpGs (%)
	Alignment efficiency (%)	n. of human CpGs^a^	Alignment efficiency (%)	Reads mapped to the human genome (%)	Reads mapped to the mouse genome (%)	n. of human CpGs^a^	n. of moue CpGs^a^	
Case_1_Normal	72.2	2,317,726	72.2	99.9	0.1	2,315,989	102	99.9
Case_1_Tumuor	74.1	2,676,050	74.1	99.9	0.1	2,674,961	119	99.9
Case_1_PDTX3	71.5	3,560,299	73.8	96.8	3.2	3,556,996	223,997	99.9
Case_2_Normal	70.6	1,893,234	70.6	99.9	0.1	1,891,949	60	99.9
Case_2_Tumuor	71.2	2,071,412	72.1	98.8	1.2	2,070,098	452	99.9
Case_2_PDTX1	68.5	2,132,270	73.4	93.2	6.8	2,130,231	90,072	99.8
Case_3_PDTX2	67.4	2,620,064	70.8	95.2	4.8	2,617,504	99,418	99.8
Case_3_PDTX3	54.7	1,623,532	71.0	76.9	23.1	1,620,101	339,859	99.7
Case_4_PDTX1	73.1	2,812,733	75.0	97.4	2.6	2,811,314	47,455	99.9
Case_4_PDTX2	69.1	1,503,423	72.3	95.6	4.4	1,501,890	13,819	99.8
Case_4_PDTX3	71.1	1,468,861	74.0	96.1	3.9	1,467,659	24,082	99.9
Case_4_PDTX5	69.2	3,185,468	73.5	94.0	6.0	3,182,452	264,898	99.9
Case_5_Tumuor	72.8	3,004,752	72.8	99.9	0.1	3,003,290	87	99.9
Case_5_PDTX3	65.8	2,066,808	70.6	93.0	7.0	2,064,469	179,031	99.8

^a^coverage > 5

The number of human CpGs having coverage higher than 5 was slightly lower when using the ICRG, but the magnitude of this effect was negligible. Indeed, we observed an average 0.06% ± 0.01 reduction in the number of CpGs in normal and tumour samples and 0.10% ± 0.04 in PDTX samples. CpG coverage was very similar independently of the reference genome used for alignment (average correlation >0.999 for normal and tumour samples as well as for PDTX samples) and similar results were obtained looking at the percentage of methylation in each human CpG (average correlation >0.999 for normal and tumour samples as well as for PDTX samples).

All together, these results suggest that the use of the ICRG for sequence alignment accurately discriminates between human and mouse reads in RRBS data. Consequently, this approach can enable the analysis of mouse stroma specific methylation signals. Indeed, in three of the PDTX samples, more than 2 × 10^^5^ mouse CpGs could be queried, a reasonable number to derive an informative methylation profile. As noted above, the number of mouse CpGs available for analysis depends on both sequencing coverage and percentage of mouse stroma in the sample.

### Using the ICRG allows dissecting expression of mouse stroma genes in PDTX-derived RNA-seq data

We tested the effect of using the ICRG in analysing PDTX RNA-seq data. First we evaluated the impact of the reference genome using the Human Reference RNA (HRR) and Mouse Reference RNA (MRR) samples for which RNA-seq data were obtained in triplicate (Table 3). In HRR samples the alignment efficiency was basically not affected by the reference genome used, although, statistically, a significant increase was observed (average efficiency = 79.05 for the HRG and 79.10 for the ICRG, paired t-test *P* = 0.019). In addition, using the ICRG, 99.8% of the reads from the HRR samples aligned to the human genome, and 98.9% of the reads from the MRR samples aligned to the mouse genome (Table 3). This reassured us that we could use the ICRG approach to distinguish between mouse and human transcripts in bulk RNA-seq data generated from PDTXs.

**Table 3:** Alignment of RNA-seq data from the human and mouse universal reference RNA

Sample	Replicate	HGR	ICRG		
		Alignment efficiency (%)	Alignment efficiency (%)	Reads mapped to the human genome (%)	Reads mapped to the mouse genome (%)
Human universal reference RNA	a	79.22	79.29	99.82	0.18
Human universal reference RNA	b	78.92	78.92	99.83	0.17
Human universal reference RNA	c	78.99	78.99	99.84	0.16
Mouse universal reference RNA	a	5.16	5.16	1.08	98.92
Mouse universal reference RNA	b	5.10	5.10	1.08	98.92
Mouse universal reference RNA	c	5.18	5.18	1.23	98.77

**Fig. 3:**
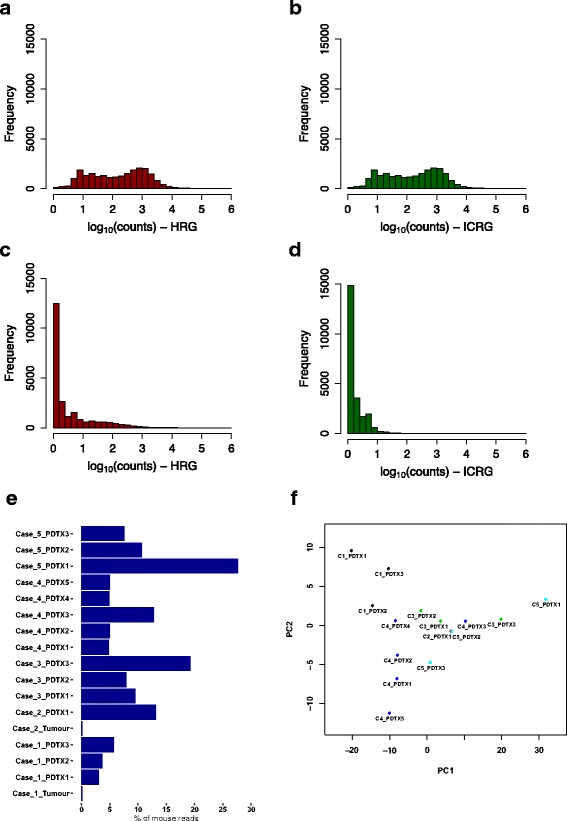
Use of the ICRG in RNA-seq data. **a**-**d** Bar plots of log_10_ transformed read counts for 23,059 human genes (having a read count higher than 5 in at least one sample). HRR sample (**a**-**b**); MRR sample (**c**-**d**). Alignment: HRG (**a**, **c**); ICRG (**b**, **d**). **e** Percentage of reads mapped to mouse in the ICRG across samples analysed. **f** Principle Component Analysis scatter plot using FPKM values of 4275 mouse genes with median FPKM > 1 (15 PDTX samples; 5 models). Different colours represent the distinct 5 PDTX models

We observed that, in the MRR sample, an average of 5.2% of the reads mapped to the human genome if the HRG was used as reference for alignment (Table 3). To evaluate the impact of these reads on the quantification of human transcripts, we computed the read counts for all human genes in the HRR and MRR samples after alignment against either the HRG or the ICRG respectively. As shown in Fig. 3a-b, in the HRR samples, gene expression quantification was not affected by the reference genome used. Indeed, the Pearson correlation between read counts in data aligned to the HRG and read counts in data aligned to the ICRG was >0.999 for all replicates. In the MRR samples, the bulk of human genes already had read count 0 or close to 0 when using the HRG for alignment (Fig. 3c). However, an average of 1565 genes (across the three MRR replicates) had read counts higher than 100, but with the use of the ICRG for alignment the number dropped to 25 genes (Fig. 3c-d). Therefore, the presence of mouse reads could introduce some bias in the quantification of a subset of human genes and the use of the ICRG in the alignment step drastically reduce this artefact.

We generated RNA-seq data for a set of matched human breast cancer samples and PDTXs (Additional file 1). As expected, in primary human samples, the percentage of reads mapped to the mouse genome was as low as 0.1%. In contrast, in the matched PDTX samples, between 3 and 27.6% of the RNA-seq reads were aligned to the mouse genome (Fig. 3e). Consequently, the use of the ICRG for alignment enables the in silico dissection of the human and mouse transcriptomes, and hence the study of gene expression signals from the human tumour cells and the mouse microenvironment in PDTXs.

The number of mouse genes detected in the PDTX samples depends on the amount of mouse stroma in the sample as well as sequencing depth. In our cohort of PDTXs (*n* = 15) sequenced to an average depth of 21 million reads per sample, 4275 mouse genes had a FPKM >1 in at least 50% of the samples. Not surprisingly, fibroblast and extracellular matrix specific genes like *Sparc*, *Bgn*, cathepsins and collagens were among the top 50 most expressed mouse genes (Additional file 2: Table S2). Interestingly, in an unsupervised Principle Component Analysis using mouse gene expression values, different passages of the same PDTX model tended to cluster together, and apart from the other models [except for one outlier sample: C3_PDTX3] (Fig. 3f).

### Comparison with other methods

As a final step, we wanted to compare our approach with previously described methods, in particular Disambiguate [12] and Xenome [11]. For this comparison, we selected the 100% human DNA samples and 100% mouse DNA samples WES data, as well as the HRR and MRR RNA-seq data.

WES data were aligned using BWA, compatible with all tested tools. We looked in particular at the total number of reads mapped to the correct genome and having MAPQ score > 0. As reported in Table 4, the ICRG and Disambiguate showed very comparable performances while the ICRG slightly outperformed Xenome, mapping 0.6% more reads to the human genome in the 100% human DNA samples and 2% more reads to the mouse genome in the 100% mouse DNA samples.

**Table 4:** Comparison of the number of reads assigned to the human and mouse genome using the ICRG, Disambiguate or Xenome

				ICRG		Disambiguate			Xenome	
Data type	% human	% mouse	Replicate	Reads mapped as human	Reads mapped as mouse	Reads mapped as human	Reads mapped as mouse	Ambiguous reads	Reads mapped as human	Reads mapped as mouse
WES										
	100	0	a	*58,106,275*	14,895	*58,109,701*	11,301	110,603	*57,764,467*	10,471
	100	0	b	*39,554,955*	12,132	*39,557,693*	9281	97,294	*39,298,032*	8847
	100	0	c	*25,372,704*	7216	*25,374,460*	5414	67,498	*25,204,254*	5359
	0	100	a	41,454	*32,542,045*	73,117	*32,497,314*	364,417	41,821	*31,900,526*
	0	100	b	39,132	*31,217,211*	70,693	*31,173,450*	366,702	39,238	*30,586,007*
	0	100	c	49,731	*44,526,608*	90,060	*44,470,789*	455,206	49,964	*43,674,812*
RNA-seq										
	100	0	a	*53,106,950*	212,696	*53,021,136*	242,026	181,712	*50,992,384*	86,370
	100	0	b	*56,450,410*	232,876	*56,364,238*	260,810	212,462	*54,219,538*	84,236
	100	0	c	*46,946,248*	206,112	*46,856,870*	220,970	199,938	*45,137,450*	64,950
	0	100	a	1,510,196	*39,721,816*	1,353,926	*39,543,932*	428,688	875,920	*38,259,766*
	0	100	b	1,240,462	*38,081,386*	1,127,872	*37,942,438*	342,720	737,946	*36,710,860*
	0	100	c	1,611,790	*44,442,932*	1,457,070	*44,267,370*	446,048	956,948	*42,837,414*

values in italic indicate the number of reads mapped to the correct genome

We also compared the CPU time required in an alignment pipeline that included either the ICRG, Disambiguate or Xenome. This was tested in the 90% human and 10% mouse DNA samples since they best approximate a real scenario. The three alignment pipelines are described in Additional file 3. The use of Xenome pipeline required 3–8% more CPU time than the ICRG and the use of Disambiguate required 35–42% more CPU time than the ICRG (Table 5).

**Table 5:** Comparison of the CPU time required by a WES alignment pipeline including either the ICRG, Disambiguate or Xenome

			CPU Time (s)		
%human	%mouse	Replicate	ICRG	Disambiguate	Xenome
90	10	a	20,154	28,743	20,905
90	10	b	20,614	28,034	22,279

An alignment pipeline using STAR and either the ICRG, Disambiguate or Xenome was applied to the HRR and MRR RNA-seq samples. As before, we focused on the total number of reads that each method was able to align to the correct genome. Also in RNA-seq data the ICRG and Disambiguate showed equivalent performances while the ICRG mapped to the correct genome an average of 4% more reads than Xenome (Table 4).

Overall, the comparison with existing methods to discriminate reads from two different species highlighted that our approach achieved the same performance as Disambiguate but was significantly faster and outperformed Xenome in terms of accuracy.

## Discussion

The use of PDTXs as preclinical models is growing exponentially because they better resemble clinical tumours compared with cell lines. They are becoming the model of choice to study tumour progression and evolution, heterogeneity and pharmacogenomics [2, 13]. At the same time, sequencing based technology has become the standard for cancer molecular characterization at the genomic, transcriptomic and epigenomic levels [14–16]. As previously suggested [11, 17], we show that standard approaches for HTS data analysis based on the alignment of raw data to the human genome can significantly compromise results and data interpretation. The use of a combined reference genome has been informally suggested in the open source community, but we demonstrate here that the alignment to the ICRG is a simple and effective strategy to distinguish between human and mouse reads in PDTX samples, preventing the identification of hundreds of false positive mutations in WES data and enabling the study of transcriptomes and methylomes of both human cancer cells and mouse stroma.

For WES data, we developed a model able to predict the percentage of human/mouse DNA content in independent samples. We applied an earlier version of this model to a cohort of breast cancer PDTXs where the average mouse stroma content was 15% [2]. Such amount of mouse stroma is enough to generate hundreds of false positive mutations if the human reference genome is used for alignment. After alignment to the ICRG, some PDTX specific mutations (i.e. present in the PDTX but not in the matched clinical tumour) were still detected. Importantly, we excluded that these were caused by misaligned mouse reads. PDTX specific mutations have several explanations: spatial heterogeneity in the donor tumour, clonal selection/evolution upon engraftment [1, 2], coverage discrepancies between the human tumour sample and the PDTX, or false positive calls.

One of the solutions adopted in previous studies to limit the high false positive rate caused by misaligned mouse reads, was to obtain Whole Genome Sequencing data for the host mouse and mask all SNVs called after mapping the data against the human genome [1]. Although the method is valid, extra sequencing data need to be obtained and extra analyses need to be run. Moreover, the presence of masked regions (>2 × 10^6^ SNVs) will increase the false negative rate.

The impact of mouse reads in RNA-seq data seems to be significantly smaller, however we still suggest aligning the data to the ICRG to avoid any bias. Moreover, this approach enables an in silico dissection of the tumour (human) and microenvironment (mouse) expression profiles. Obviously, the amount of genes that can be quantified in the mouse compartment depends on both the amount of stromal infiltration (biological variable that we probably want to capture) and the sequencing depth coverage (technical variable that we want to minimise). We therefore recommend that a higher and uniform number of reads is obtained in PDTX RNA-seq experiments. Using a sequencing depth of 21 million reads and with an average mouse read percentage of 8.5%, we found more than 4000 mouse genes with FPKM > 1 in at least 50% of the PDTX samples. Unsupervised analysis of these expression profiles grouped together different passages from the same model, suggesting that each PDTX model induces specific transcriptomic changes in the mouse microenvironment that can be explored using the ICRG approach. Our RNA-seq libraries were sequenced using the HiSeq 4000 Illumina instrument that has been reported to be affected by ‘index hopping’, consisting in around 1% of the reads being assigned to the wrong barcode (i.e. sample) [18]. Although this is unlikely to have a tangible impact in our experimental setting, some of the reads aligned to the wrong genome could be explained by this phenomenon.

It was reassuring to observe that ICRG alignment performed well with RRBS data. In this data type, the bisulphite treatment of samples will convert methylated cytosine bases to thymine and then for downstream analysis all cytosine bases are converted in silico to thymine for alignment purposes (three letter aligners) [19], reducing read complexity and, consequently, making multiple mapping or misalignment more likely. Although, similarly to RNA-seq data, the use of the ICRG for alignment is not strictly required, we would still recommend it since it enables the methylome profiling of the mouse stroma.

An important aspect of this work is that the experiments generated using controlled dilutions represent a relevant benchmark dataset for further investigations. All sequencing data generated in this study are available through the European Genome-Phenome Archive (EGA, https://ega-archive.org/) under accession number EGAD00001003800.

Importantly, we compared our method with previously reported methods, namely Disambiguate [12] and Xenome [11]. Our method was able to recover a higher number of reads mapped to the correct genome than Xenome, while showing a comparable performance with Disambiguate. However, an alignment pipeline using the latter required around 40% more time to complete, a significant difference for what is the most time-consuming step in the analysis of HTS data. Moreover, the implementation of an ICRG-based pipeline is compatible with any alignment software and does not require any extra software to be installed and incorporated, but only the ‘one-off’ generation of aligner indices. To facilitate a smooth implementation of our method, all the relevant code is available at https://github.com/cclab-brca/ICRG.

## Conclusions

In conclusion, we present here a straightforward strategy, based on the use of ICRG for read alignment, which is able to handle the presence of mouse reads in PDTX sequencing data. We demonstrate that this approach is efficient in removing mouse reads before performing somatic mutation calling and that it allows estimation of the human/mouse DNA content in the xenograft sample. In addition, the use of the ICRG enables human tumour and mouse stroma specific analysis of transcriptome and methylome profiles. In a direct comparison with previously reported methods we observed similar or higher performances in terms of accuracy and a significantly reduced computational time.

## Methods

### Sample description

We used a surgical tumour sample and mouse mammary fat pad as a source of pure human and mouse tissue. In this study, we also included 5 breast cancer cases and their matched PDTXs (Additional file 1) that are part of our previously reported biobank [2]. Signed consent was obtained from the patients whose tumour samples were used in this study and all research was conducted with the appropriate approval by the National Research Ethics Service [Cambridgeshire 2 REC reference number: 08/H0308/178]. Mice were bought from Charles River^®^. Animals were euthanised by cervical dislocation and death confirmed by a secondary method according to Schedule 1 of the Scientific Procedure Act (1986). Tumour tissue was removed in aseptic conditions and all animal experiments were conducted in compliance with the rigorous Home Office framework of regulations (Project License 707,679).

Pure human and mouse reference RNAs were purchased: Universal Human Reference RNA (HRR, Agilent Technologies Inc., USA, 740,000); and Universal Mouse Reference RNA (MRR, Agilent Technologies Inc., USA, 740,100).

### Nucleic acid purification

DNA was extracted from all samples using the Qiagen Blood and Tissue kit (Cat ID, 69,504) as per manufacturer’s instructions. To generate a human-mouse DNA dilution series, human and mouse pure DNA concentration was normalised and then mixed in predefined proportions volumetrically.

RNA was extracted from all samples using the Qiagen miRNeasy kit (Cat ID, 217,004) as per manufacturer’s instructions.

### Reference genomes for read alignment

Two reference genomes were used in our study. The first was the standard Human Reference Genome (hg19/GRCh37 decoy) hereafter called HRG. The second was the ICRG, generated by combining the aforementioned HRG with the mouse reference genome (mm10). Mouse chromosomes were renamed as “m.chr” and then the two fasta files (human and mouse) were concatenated. The concatenated fasta file was then indexed using the appropriate tool provided by each aligner.

### Whole exome sequencing

WES libraries were prepared using Nextera Rapid Capture Exome (Illumina Inc., USA) following manufacturer’s instructions [2]. Sequencing was performed using 75 bp paired-end reads for the human/mouse dilution series and 125 bp paired-end reads for human and PDTX samples. Demultiplexing was performed using bcl2fastq2 v.2.17 software allowing 0 mismatches. Sequencing quality of raw fastq files was assessed using FastQC (v 0.11.5, http://www.bioinformatics.babraham.ac.uk/projects/fastqc).

Raw data were processed according to the ITC approach described in [20]. Briefly, alignment was performed using BWA-MEM (v 0.7.12) and Novoalign (v 3.02) followed by mutation calling with Mutect2 and Strelka. Only the intersection of mutations called by the same caller after different alignment were retained. Then, mutations called by the two callers were merged to generate the final set of identified somatic mutations (SNVs and Indels). Alignment efficiency (i.e. the percentage of reads that aligned to the reference sequence) and statistics were derived from Novoalign-aligned bam files using Picard Tools (v 1.140) or custom scripts (https://github.com/cclab-brca/ICRG). The same pipeline was applied using either the HRG or the ICRG in the alignment step. For each PDTX-clinical tumour pair, the variant allele frequencies (VAFs) of the mutations called in at least one sample were re-computed in both samples using GATK HaplotypeCaller (v 3.5). If the VAF was >1% in both samples the mutation was defined as common, otherwise it was defined as either tumour specific or PDTX specific.

In WES data from the human-mouse DNA dilution series aligned using the ICRG, we used custom bash code to compute the percentage of reads mapped to the human genome. Using the R package mgcv [21] these values and the known percentage of human DNA in the sample were used to derive a calibration curve applying penalized regression splines with a basis dimension of 3.

### RNA-sequencing

Libraries for Illumina sequencing were prepared using TruSeq Stranded mRNA HT kit (Cat ID, RS-122-2103, Illumina). 500 ng of total RNA with RNA Integrity Numbers (RINs) above 8 was used for library preparation. Samples were processed following manufacturer’s HS (High-Sample) instructions (part# 15031048 Rev. E, Illumina) with 12 cycles of PCR used at the Enrichment of DNA Fragments step. All libraries were quantified using KAPA Library Quantification Kit Illumina ROX Low (Cat ID, KK4873, KAPA Biosystems) and normalised. Libraries were pooled in equal volumes and pools were used for clustering on HiSeq4000 sequencing flow cell following manufacturer’s instructions. Sequencing was performed using 150 bp paired-end run type for dual-indexed libraries.

Demultiplexing was performed using bcl2fastq2 v.2.17 software allowing 0 mismatches. Sequencing quality of raw fastq files was assessed using FastQC (v 0.11.5, http://www.bioinformatics.babraham.ac.uk/projects/fastqc) and alignment to HRG or ICRG was performed using STAR v2.5.2 in two-pass mode for splice-aware read alignment [22]. The resulting BAM file was then assessed using RNASeQC (v1.1.8) [23].

Counting of reads aligned over exonic features for the purpose of gene expression quantification was performed using the htseq-count script in the HTSeq package (v 0.6.1) in ‘Union’ overlap resolution mode [24]. The Gene Transfer Format (GTF) file used for the purposes of counting was a merged *Homo sapiens* and *Mus musculus* GTF file, both obtained from Ensembl (http://www.ensembl.org), and modified to ensure chromosomal compatibility with the ICRG. The resulting counts for all samples were then collated and FPKM calculations per gene per sample were performed using the rpkm() function in the edgeR R package [25].

### RRBS

DNA was quantified using Quant-iT High Sensitivity dsDNA Assay (Thermo Fisher, USA) and 200 ng was used as input for RRBS library preparation. DNA was subjected to an optimised protocol [26] and pooled prior to bisulphite conversion using Zymo Research EZ DNA Methylation gold kit (Cat ID, D5006). Pooled bisulphite converted samples were amplified with 15 cycles of PCR and purified twice with SPRI beads (Agencourt AMPure XP, Beckman Coulter, Cat ID A63880) for size selection, using 2X then 1.5X volume of the elute. Libraries were assessed for concentration and quality respectively using qPCR (KAPA Biosystems, KK4873) and DNA High sensitivity chip on Bioanalyser 2100 (Agilent Technologies Inc., USA). RRBS sequencing was performed using 125 bp paired-end reads. Demultiplexing was performed using bcl2fastq2 v.2.17 software allowing 0 mismatches. Sequencing quality of raw fastq files was assessed using FastQC (v 0.11.5, http://www.bioinformatics.babraham.ac.uk/projects/fastqc). Bismark (version 0.13.1) was used for read alignment and to derive alignment stats. Only CpGs with at least 5× coverage were selected for subsequent analysis. The pipeline was run twice, using HRG and ICRG, respectively.

### Comparison with other methods

In the comparison analysis, alignment was performed using BWA-MEM (v 0.7.12) for WES data and STAR v2.5.2 for RNA-seq data. Our approach was compared to Disambiguate [12] (C++ version downloaded from https://github.com/AstraZeneca-NGS/disambiguate) and Xenome [11] included in the Gossamer bioinformatics suite (https://github.com/data61/gossamer). The number of reads mapping to each genome was computed using samtools and the grep function as detailed in the code available at https://github.com/cclab-brca/ICRG. For the 90% human and 10% mouse DNA samples, the CPU time was extracted from the pipeline log file and a pipeline description for each method is reported in Additional file 2.

### Other analyses

Bam files were visualized using IGV (http://software.broadinstitute.org/software/igv/). Processed data mining and graphical representation of the results were performed using R/Bioconductor (v 3.2.2).

## Additional files

Additional file 1: Molecular data generated - Table detailing which molecular data (WES, RNA-seq or RRBS) were generated for each sample included in the study. (XLSX 39 kb)

Additional file 2: WES alignment pipelines including either the ICRG, Disambiguate or Xenome - Schematic representation of the WES alignment pipelines developed to compare the ICRG method with Disambiguate and Xenome. (PPTX 41 kb)

## Abbreviations

HRG: Human Reference Genome
HRR: Human Reference RNA
HTS: High Throughput Sequencing
ICRG: In silico Combined human-mouse Reference Genome
MRR: Mouse Reference RNA
PDTX: Patient-Derived Tumour Xenograft
RRBS: Reduced Representation Bisulfite Sequencing
SNV: Single Nucleotide Variant
VAF: Variant Allele Frequency
WES: Whole Exome Sequencing

## References

[1] Eirew P, Steif A, Khattra J, Ha G, Yap D, Farahani H (2015). Dynamics of genomic clones in breast cancer patient xenografts at single-cell resolution. Nature.

[2] Bruna A, Rueda OM, Greenwood W, Batra AS, Callari M, Batra RN, et al. A biobank of breast cancer explants with preserved intra-tumor heterogeneity to screen anticancer compounds. Cell. 2016; 10.1016/j.cell.2016.08.041. 10.1016/j.cell.2016.08.041 PMC5037319 27641504

[3] Gao H, Korn JM, Ferretti S, Monahan JE, Wang Y, Singh M (2015). High-throughput screening using patient-derived tumor xenografts to predict clinical trial drug response. Nat Med.

[4] Hidalgo M, Amant F, Biankin AV, Budinska E, Byrne AT, Caldas C (2014). Patient-derived Xenograft models: an emerging platform for translational cancer research. Cancer Discov.

[5] Cassidy JW, Batra AS, Greenwood W, Bruna A (2016). Patient-derived tumour xenografts for breast cancer drug discovery. Endocr Relat Cancer.

[6] Ocana A, Pandiella A, Siu LL, Tannock IF (2011). Preclinical development of molecular-targeted agents for cancer. Nat Rev Clin Oncol.

[7] Tentler JJ, Tan AC, Weekes CD, Jimeno A, Leong S, Pitts TM (2012). Patient-derived tumour xenografts as models for oncology drug development. Nat Rev Clin Oncol.

[8] Byrne AT, Alferez DG, Amant F, Annibali D, Arribas J, Biankin AV (2017). Interrogating open issues in cancer precision medicine with patient-derived xenografts. Nat Rev Cancer.

[9] DeRose YS, Wang G, Lin Y-C, Bernard PS, Buys SS, Ebbert MTW (2011). Tumor grafts derived from women with breast cancer authentically reflect tumor pathology, growth, metastasis and disease outcomes. Nat Med.

[10] Reyal F, Guyader C, Decraene C, Lucchesi C, Auger N, Assayag F (2012). Molecular profiling of patient-derived breast cancer xenografts. Breast Cancer Res.

[11] Conway T, Wazny J, Bromage A, Tymms M, Sooraj D, Williams ED (2012). Xenome--a tool for classifying reads from xenograft samples. Bioinformatics.

[12] Ahdesmäki MJ, Gray SR, Johnson JH, Lai Z (2016). Disambiguate: an open-source application for disambiguating two species in next generation sequencing data from grafted samples. F1000Res.

[13] Cassidy JW, Caldas C, Bruna A (2015). Maintaining tumor heterogeneity in patient-derived tumor Xenografts. Cancer Res.

[14] Lawrence MS, Stojanov P, Mermel CH, Robinson JT, Garraway LA, Golub TR (2014). Discovery and saturation analysis of cancer genes across 21 tumour types. Nature.

[15] Zack TI, Schumacher SE, Carter SL, Cherniack AD, Saksena G, Tabak B (2013). Pan-Cancer patterns of somatic copy number alteration. Nat Genet.

[16] E. Ellsworth R, J. Decewicz D, D. Shriver C, L. Ellsworth D. Breast Cancer In the personal genomics era. Curr Genomics 2010;11:146–161. doi:10.2174/138920210791110951. 10.2174/138920210791110951 PMC2878980 21037853

[17] Khandelwal G, Girotti MR, Smowton C, Taylor S, Wirth C, Dynowski M, et al. Genomics next-generation sequencing analysis and algorithms for PDX and CDX models. Mol Cancer Res. 1 10.1158/1541-7786.MCR-16-0431. 10.1158/1541-7786.MCR-16-0431 28442585

[18] Illumina. Effects of index Misassignment on multiplexing and downstream analysis. https://www.illumina.com/content/dam/illumina-marketing/documents/products/whitepapers/index-hopping-white-paper-770-2017-004.pdf?linkId=36607862. Accessed 16 Oct 2017.

[19] Krueger F, Andrews SR (2011). Bismark: a flexible aligner and methylation caller for Bisulfite-Seq applications. Bioinformatics.

[20] Callari M, Sammut S-J, De Mattos-Arruda L, Bruna A, Rueda OM, Chin S-F (2017). Intersect-then-combine approach: improving the performance of somatic variant calling in whole exome sequencing data using multiple aligners and callers. Genome Med.

[21] Wood SN. Generalized additive models: an introduction with Boca Raton: R: Chapman & Hall/CRC; 2006.

[22] Dobin A, Davis CA, Schlesinger F, Drenkow J, Zaleski C, Jha S (2013). STAR: ultrafast universal RNA-seq aligner. Bioinformatics.

[23] DeLuca DS, Levin JZ, Sivachenko A, Fennell T, Nazaire M-D, Williams C (2012). RNA-SeQC: RNA-seq metrics for quality control and process optimization. Bioinformatics.

[24] Anders S, Pyl PT, Huber W (2015). HTSeq--a python framework to work with high-throughput sequencing data. Bioinformatics.

[25] Robinson MD, McCarthy DJ, Smyth GK (2010). edgeR: a bioconductor package for differential expression analysis of digital gene expression data. Bioinforma.

[26] Tufegdzic Vidakovic A, Rueda OM, Vervoort SJ, Sati Batra A, Goldgraben MA, Uribe-Lewis S (2015). Context-specific effects of TGF-β/SMAD3 in cancer are modulated by the Epigenome. Cell Rep.

