# Supplementary material for: Computational approach to discriminate human and mouse sequences in patient-derived tumour xenografts
Source: BMC Genomics. 2018 Jan 5;19:19. doi: 10.1186/s12864-017-4414-y (PMC5755132; doi:10.1186/s12864-017-4414-y)
Supplement: Supplementary file 2 — WES alignment pipelines including either the ICRG, Disambiguate or Xenome - Schematic representation of the WES alignment pipelines developed to compare the ICRG method with Disambiguate and Xenome. (PPTX 41 kb) [file 12864_2017_4414_MOESM2_ESM.pptx]

## Slide 1
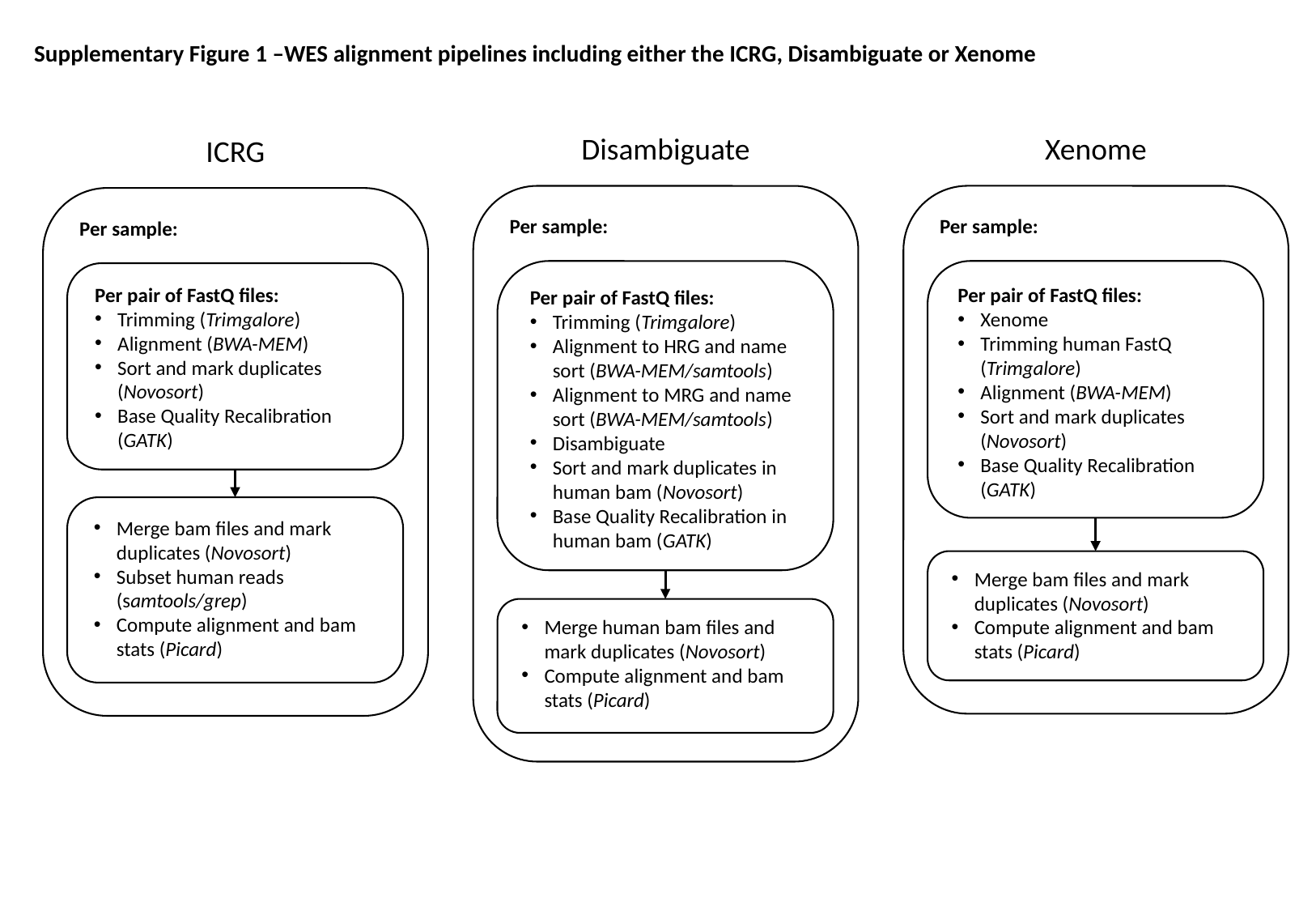

Supplementary Figure 1 –WES alignment pipelines including either the ICRG, Disambiguate or Xenome
Disambiguate
Xenome
ICRG
Per sample:
Per pair of FastQ files:
Trimming (Trimgalore)
Alignment to HRG and name sort (BWA-MEM/samtools)
Alignment to MRG and name sort (BWA-MEM/samtools)
Disambiguate
Sort and mark duplicates in human bam (Novosort)
Base Quality Recalibration in human bam (GATK)
Merge human bam files and mark duplicates (Novosort)
Compute alignment and bam stats (Picard)
Per sample:
Per pair of FastQ files:
Xenome
Trimming human FastQ (Trimgalore)
Alignment (BWA-MEM)
Sort and mark duplicates (Novosort)
Base Quality Recalibration (GATK)
Merge bam files and mark duplicates (Novosort)
Compute alignment and bam stats (Picard)
Per sample:
Per pair of FastQ files:
Trimming (Trimgalore)
Alignment (BWA-MEM)
Sort and mark duplicates (Novosort)
Base Quality Recalibration (GATK)
Merge bam files and mark duplicates (Novosort)
Subset human reads (samtools/grep)
Compute alignment and bam stats (Picard)
